# MicroRNA and Microvascular Complications of Diabetes

**DOI:** 10.1155/2018/6890501

**Published:** 2018-03-07

**Authors:** F. Barutta, S. Bellini, R. Mastrocola, G. Bruno, G. Gruden

**Affiliations:** ^1^Laboratory of Diabetic Nephropathy, Department of Medical Sciences, University of Turin, Turin, Italy; ^2^Department of Clinical and Biological Sciences, University of Turin, Turin, Italy

## Abstract

In the last decade, miRNAs have received substantial attention as potential players of diabetes microvascular complications, affecting the kidney, the retina, and the peripheral neurons. Compelling evidence indicates that abnormally expressed miRNAs have pivotal roles in key pathogenic processes of microvascular complications, such as fibrosis, apoptosis, inflammation, and angiogenesis. Moreover, clinical research into innovative both diagnostic and prognostic tools suggests circulating miRNAs as possible novel noninvasive markers of diabetes microvascular complications. In this review, we summarize current knowledge and understanding of the role of miRNAs in the injury to the microvascular bed in diabetes and discuss the potential of miRNAs as clinical biomarkers of diabetes microvascular complications.

## 1. Introduction

Microvascular complications of diabetes (DMC) have a significant impact on quality of life, morbidity, and mortality, posing a huge burden on the health care system. Diabetic nephropathy is a leading cause of end-stage renal disease (ESRD) and augments the risk of cardiovascular diseases (CVD). Diabetic retinopathy is the major cause of new blindness in adults, and diabetic neuropathy contributes to the development of foot ulcerations that are the predominant cause of nontraumatic lower limb amputation among adults. Therefore, it is urgent to identify novel targets for treatment and to discover innovative noninvasive biomarkers to improve risk prediction, early diagnosis, and prognosis assessment.

MicroRNAs (miRNAs) have been recently implicated in a growing number of pathophysiological conditions. Moreover, miRNAs are also present in biological fluids in a stable form and have been proposed as noninvasive biomarkers. Recently, a growing number of basic science studies have demonstrated the key role of miRNAs in the pathogenesis of DMC. In addition, clinical studies have provided preliminary evidence that miRNAs present in body fluids may be exploited as biomarkers of DMC.

This review summarizes current knowledge on miRNAs as both pathogenic mechanisms and biomarkers of DMC. Furthermore, we will discuss potential future perspectives and limits of this novel line of research.

## 2. MicroRNAs

MiRNAs are small (~22 nucleotides) noncoding RNA sequences that inhibit gene expression of specific mRNA targets. MiRNAs are present in mammalians, plants, and some viruses. Over 2500 mature miRNAs have been identified in the human genome. Each of them regulates the expression of multiple genes and single mRNAs are targeted by multiple miRNAs; therefore, miRNAs are involved in a vast array of pathophysiological processes [1].

### 2.1. MicroRNA Synthesis

The first step in miRNA synthesis is the transcription of miRNA genes by RNA polymerase II into large RNA precursors called pri-miRNAs, containing a 5′ cap and a poly-A tail [2]. As multiple miRNAs can be transcribed from the same gene, pri-miRNAs can give rise to various mature miRNAs. Pri-miRNAs are processed in the nucleus by the microprocessor complex, containing the RNase III Drosha that cuts pri-miRNAs and releases stem-loop sequences of 60–70 nucleotides called pre-miRNAs [3]. The pre-miRNA is transferred from the nuclei to the cytosol by the exportin-5 and Ran-GTP complex [4] In the cytosol, the RNase III Dicer processes pre-miRNAs with formation of duplex miRNAs (dsmiRNA), containing a guide and a passenger strand. dsmiRNAs form an intermediate complex, named miR-RISC (RNA-induced silencing complex), including Dicer and both Argonaute (Ago) and TRBP proteins. During this step, the passenger strand is removed and the mature single-strand miRNA binds to Ago within a functional RISC complex [5–8].

### 2.2. MicroRNA Function

Mature miRNAs act as a guide allowing the RISC to recognize complementary sequences at the 3′ untranslated region (3′ UTR) of their target mRNAs. In most cases, the recognition occurs between the target mRNA and a sequence of 2–8 bases of the miRNA [9]. The predominant mechanism whereby miRNAs negatively control the expression of their targets is translational repression and subsequent degradation of target mRNAs. Translational repression is usually followed by mRNA degradation; however, this is not always the case and a repressed mRNA may undergo translational reactivation. Degradation may occur through different mechanisms. Ago proteins possess nuclease activity and may directly degrade mRNAs. Alternatively, Ago can function as a binding site for TNRC6-A/B/C proteins that promote mRNA deadenylation. Following deadenylation, mRNAs are rapidly degraded by 3′–5′ exonucleases [10].

## 3. Diabetes Microvascular Complications

### 3.1. Diabetic Retinopathy

In the developed countries, diabetic retinopathy (DR) is the leading cause of new blindness in people aged 25–74 years. DR prevalence is strongly related to both level of glycemic control and diabetes duration, and intervention studies have convincingly demonstrated the importance of hyperglycemia in the rate of both development and progression of DR. There are two major DR stages: nonproliferative (NPDR) and proliferative (PDR) retinopathy. In NPDR, thickening of the basement membrane and pericytes/endothelial cell loss result in increased vascular permeability and development of microvascular abnormalities, such as dilated vessels, capillary microaneurysms, shunts, and vascular occlusion. In the severe NPDR stage, vessel obliteration deprives blood supply to areas of the retina that secrete factors to promote formation of new blood vessels. In PDR, both leaking and breaking of immature and fragile new vessels can cause vitreous hemorrhages, macula edema, fibrosis, retinal detachment, and possibly sight loss. A low-grade inflammation is believed to contribute to both DR stages by amplifying hyperglycemia-induced injury and partially mediating neovascularization [11].

### 3.2. Diabetic Neuropathy

Diabetic neuropathies are the most common complications of DM, affecting as many as 50% of patients. Distal symmetric polyneuropathy (DSPN), the most prevalent form of diabetic neuropathy, is a chronic, symmetrical, length-dependent sensorimotor polyneuropathy. DSPN develops after long-standing hyperglycemia and can be stabilized by rigorous glycemic control. The complication is due to both metabolic derangement, directly damaging the neurons, and injury of the small blood vessels supplying the nerves. Axonal degeneration is the primary structural abnormality; however, demyelination resulting from Schwann cell dysfunction also occurs. DSPN affects predominantly sensory neurons and symptoms vary according to the class of sensory fibers involved. Early symptoms due to the involvement of small fibers include pain and dysesthesias, while anesthesia and poor balance develop at later stages [12].

### 3.3. Diabetic Nephropathy

Diabetic nephropathy (DN) develops in almost a third of patients with diabetes and accounts for 44% of incident ESRD in the United States. Intervention studies in humans have demonstrated the key pathogenic role of both hyperglycemia and hypertension in DN both onset and progression. DN is characterized by increased glomerular permeability to proteins and progressive renal function decline. Injury and loss of glomerular podocytes, thickening of the glomerular basement membrane, mesangial expansion, and tubule-interstitial fibrosis are the predominant structural abnormalities. Podocyte dysfunction/damage is the major cause of albuminuria development, while excessive extracellular matrix (ECM) deposition leading to sclerosis is a key determinant of progressive renal function loss [13, 14].

## 4. MicroRNAs as Cellular Mediator of DMC

Several studies have assessed the expression of miRNAs in the tissues/cells from target organs of DMC, providing evidence that miRNA expression profile is altered in both human and experimental DMC (Table 1). However, these studies have used nondiabetic controls as the comparator group, and thus diabetes itself may account for part of the observed changes.

miRNA expression profiling can facilitate the discovery of miRNAs with a key role in DMC and also identify “molecular miRNA signatures” associated with DMC. However, as shown in Table 1, the reproducibility of the results obtained in miRNA profiling studies is scarce even when only highly differentially expressed and PCR-validated miRNAs are considered. The presence of a myriad of potential confounders, including preanalytic and analytic variables, may partially account for this. Regardless of the cause, only part of miRNAs that were differentially expressed in profiling studies was found to be important in subsequent dedicated analyses, and most of the available data come from hypothesis-driven studies, assessing selected candidate miRNAs.

In the next sections, we will review evidence for an involvement of selected miRNAs in common pathophysiological processes across DMC: angiogenesis, inflammation, cell injury/apoptosis, and fibrosis. A list and description of the most relevant miRNAs by complication are reported in Tables 2–4.

### 4.1. MicroRNAs and Neovascularization in DMC

As mentioned above, hypoxia-induced abnormal neovascularization is a characteristic feature of DR. The cytokine vascular endothelial growth factor (VEGF) plays a key role in this process and anti-VEGF therapies have been proven effective for the treatment of both diabetic macular edema and PDR. Therefore, miRNAs that control VEGF signaling have been extensively studied in the context of DR. Among them, miR-126, miR-106, miR-15, and miR-200b directly target VEGF, while miR-150, miR-184, and miR-155 indirectly modulate VEGF either expression or signaling.

*miR-126* is one of most studied miRNA in both diabetes and diabetes complications because of its key role in endothelial protection and angiogenesis. Levels of miR-126 are reduced in the retina in experimental diabetes and other hypoxic conditions [15], and there is evidence from studies in animal models of oxygen-induced retinopathy (OIR) of a causal link between hypoxia-induced miR-126 downregulation and the rise in retinal VEGF levels. For instance, intravitreous injection of a plasmid vector expressing miR-126 reduced both VEGF and neovascularization [16]. Of interest, a recent study has shown that treatment with Niaspan, which normalized retinal miR-126 levels, prevented overexpression of VEGF as well as edema, hemorrhages, and apoptosis [17]. Moreover, a small case-control study also reported an association between a miR-126 polymorphism and both severe NPDR and PDR in patients with type 2 diabetes [18]. There is interdependence between hypoxia-induced factor 1*α* (HIF1-*α*) and VEGF expression as silencing of HIF1-*α* resulted in a significant reduction in VEGF protein levels and vice versa in both *in vitro* and *in vivo* models of DR. This interdependence is mediated by shared miRNAs, such as *miR-106*, and overexpression of miR-106a significantly reduced the expression of both HIF1-*α* and VEGF and prevented high glucose-induced increased permeability [19].

McArthur et al. showed a downregulation of *miR-200b* in both experimental and human DR, and manipulation of miR-200b levels confirmed that retinal VEGF expression was under miR-200b control. Most importantly, they demonstrated that a miR-200b mimic normalizes retinal VEGF levels and reduces both neovascularization and enhanced permeability in DM mice, providing evidence that miR-200b is a key mediator of VEGF rise in DR and a potential target for miRNA-based treatment [20]. Similarly, retinal *miR-15* was reduced in both experimental and human DR, and overexpression of miR-15 reduced VEGF and ameliorated vascular leakage in DM animals. However, miR-15 has also anti-inflammatory properties and controls the expression of acid sphingomyelinase, the central enzyme in the sphingolipid metabolism; therefore, amelioration of inflammation and lipotoxicity is a possible additional protective mechanism [21].

MicroRNA-150, miR-184, and miR-155 can indirectly modulate VEGF signaling in DR. *miR-150* affects VEGF by inhibiting the expression of the type 2 VEGF receptor. This miRNA is of relevance in DR as miR-150 is downregulated in the diabetic retina [22] and miR-150 deletion exacerbated retinal neovascularization in a model of high-fat diet-induced diabetes [23], likely by further enhancing VEGF signaling. *miR-184* was downregulated in a model of ischemia-induced retinal neovascularization [24]. Because miR-184 controls the expression of frizzled-7, downregulation of miR-184 can activate the canonical Wnt/frizzled-7 pathway that plays a central role in DR by enhancing neovascularization through increased VEGF transcription [25]. Expression of *miR-155* is induced by VEGF, and this is a mechanism whereby VEGF enhances its downstream signaling. In fact, miR-155 inhibits SHIP1, a counterregulator of the VEGF-induced phosphoinositide 3-kinase (PI3K)/Akt pathway. Moreover, studies in DR have shown that retinal levels of miR-155 are enhanced in a model of OIR and that treatment with a miR-155 antagomir can reduce neovessel formation in a model of OIR via a SHIP1/PI3K/Akt-dependent pathway [26].

Collectively, these data highlight the importance of miRNAs in modulating VEGF activity and thus retinal neovascularization in DR and suggest that miRNAs may represent novel targets for treatment. However, miRNAs affecting VEGF in the diabetic retina have many other target genes, increasing the likelihood of off-target effects.

### 4.2. MicroRNAs and Inflammation in DMC

A low-grade chronic inflammation is believed to contribute to the pathogenesis of DMC. Hyperglycemia and both hemodynamic and oxidative stress are the main inducers of the inflammatory response in DMC through activation of the transcription factor NF-*κ*B. Monocyte chemoattractant protein 1 (MCP-1) and other chemokines locally recruit monocyte/macrophages that are predominantly of the M1 proinflammatory phenotype. Both resident cells and infiltrating macrophages release inflammatory cytokines that contribute to cell damage and promote inflammation-driven fibrosis. Among the multitude of miRNAs implicated in the regulation of inflammatory processes, miR-146, miR21, and miR-29 appear of particular relevance in DMC.

*miR-146a* is a well-known modulator of both the innate and adaptive immune response. In particular, miR-146 induction is a mechanism whereby NF-*κ*B limits its proinflammatory activity. In fact, NF-*κ*B activates miR-146a, which in turn inhibits NF-*κ*B by downregulating interleukin-1 receptor-associated kinase 1/2 (IRAK1/2) and TNF receptor-associated factor 6 (TRAF6). Therefore, miR-146a is crucial to allow the timely resolution of ongoing inflammatory processes.

**Figure 1:**
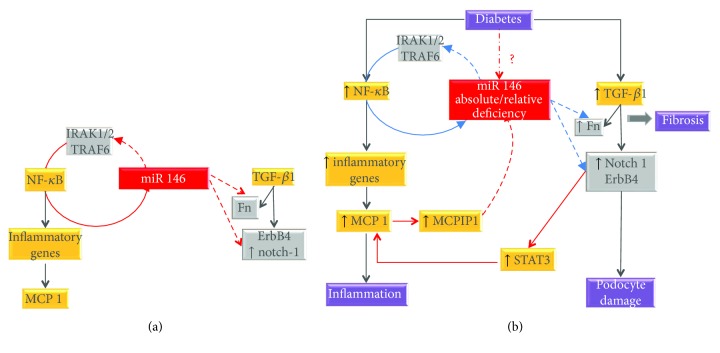
Role of miR-146a in diabetic microvascular complications. (a) In normoglycemic conditions, miR-146 is induced by NF-*κ*B, and it inhibits NF-*κ*B by suppressing its target genes interleukin-1 receptor-associated kinase 1/2 (IRAK1/2) and TNF receptor-associated factor 6 (TRAF6). Moreover, miR-146 represses expression of fibronectin (Fn) in retinal cells and ErbB4/Notch1 in podocytes. (b) In the presence of diabetes, there is an absolute/relative miR-146 deficiency leading to insufficient inhibition (light blue lines) of IRAK1/2/TRAF6 (enhancing inflammation), Fn expression (favoring fibrosis), and TGF-*β*1/ErbB4/Notch1 signaling (leading to podocyte damage). Enhanced signaling through the TGF-*β*1-ErbB4 pathway increases autocrine synthesis of MCP-1, further reducing miR-146a levels via MCPIP1 in a feed-forward loop. Grey boxes: miR-146 target genes; dotted lines: inhibition; continuous line: activation/induction.

miR-146a expression has been studied in the kidney, retina, and sciatic nerve from both patients with DMC and experimental diabetic animals. Results are conflicting with some studies reporting an increase [27–29] and other a reduction in miR-146a expression [22, 30–33]. This likely reflects variability in the compensatory anti-inflammatory miR-146a response in various stages and models of DMC. However, the observation that both NF-*κ*B activity and inflammatory cytokine levels were elevated even when miR-146a was overexpressed suggests a state of relative miR-146a deficiency with insufficient activation of the NF-*к*B-miR-146a negative feedback loop [27]. Furthermore, a recent study in podocytes has described a feed-forward loop resulting in a progressive reduction in miR-146 expression. Specifically, MCP-1 upregulates the ribonuclease MCPIP1 that antagonizes miR-146a. This reduces miR-146 inhibition on its target gene ErbB4 and thus enhances signaling through the TGF-*β*1-ErbB4 pathway, which increases autocrine synthesis of MCP-1, further reducing miR-146a levels [33] (Figure 1).

In keeping with these data, intervention studies have consistently demonstrated an anti-inflammatory and protective effect of miR-146a in DMC. In experimental DSPN, systemic administration of a miR-146a mimic improved both functional and structural alterations of DSPN and induced a shift from M1 to M2 macrophages [31, 32]. In experimental DR, intravitreal injection of miR-146a ameliorated both microvascular leakage and retinal functional defects and reduced expression of intercellular adhesion molecule 1 (ICAM-1), a NF-*κ*B downstream gene [34]. In experimental DN, deletion of miR-146a exacerbated proteinuria, fibrosis, and macrophage infiltration and induced an M2 to M1 macrophage shift with inflammasome activation [29], while treatment with erlotinib, a pan-ErbB kinase inhibitor, ameliorates podocyte injury and albuminuria by preventing ErbB4 induction secondary to miR-146 downregulation [33].

There is relatively little information on miR-146a in human DMC; however, a miR-146a polymorphism was associated with both DN and DSPN [35, 36]. Moreover, in a recent study comparing miRNA expression in human glomeruli from patients with various kidney diseases, the expression levels of miR-146a and miR-30a, used in combination, could effectively distinguish DN from all other renal conditions except IgA nephropathy [37], suggesting a potential relevance of miR-146a in the differential diagnosis of renal diseases.

*MicroRNA-29b* is of relevance in the context of inflammation because it can reduce NF-*κ*B activity by targeting Sp-1, a transcriptional factor playing a key role in the activation of the NF-*κ*B pathway. The expression of miR-29b was reduced in experimental DN, and treatments increasing miR-29b levels reduced microalbuminuria, renal fibrosis, and Sp-1/NF-*κ*B-driven inflammation in diabetic animals [38]. However, this benefit cannot be entirely ascribed to miR-29b anti-inflammatory properties because Sp-1 can also modulate TGF-*β*1 signaling/apoptosis. miR-29 is also downregulated in both DR and DSPN [39, 40], but there are no data on a potential anti-inflammatory role of miR-29b in these complications.

*miR-21* is overexpressed in both DR and DN [22, 41–45] and contributes to the pathogenesis of DR by enhancing inflammation. Indeed, intervention studies have shown that both miR-21 deletion and intravitreal injection of a miR-21 inhibitor ameliorated retinal leakage and inflammation at least in part via upregulation of peroxisome proliferator-activated receptor-*α* (PPAR-*α*) [22, 41], which is a miR-21 target and inhibits NF-*κ*B by upregulating I*κ*B-*α*. However, PPAR-*α* has also other beneficial effects particularly on metabolism that may explain its protective effect. There are no data on miR-21 expression in DSPN; however, miR-21 is upregulated in models of peripheral neuronal injury [46], and a recent study has described an unforeseen link between miR-21 and inflammation. Injured dorsal root ganglia (DRG) neurons release exosomes enriched in miR-21 that are phagocytized by macrophages in which miR-21 promotes a proinflammatory phenotype. Therefore, both upregulation and release of miR-21 appears to play a key role in sensory neuron-macrophage communication after damage to the peripheral nerve [47]. Likely future studies will explore if a similar mechanism is at play in DMC.

### 4.3. MicroRNA and Cell Injury/Apoptosis in DMC

Cell damage eventually leading to apoptosis is a characteristic feature of DMC. Moreover, both podocytes and neurons are terminally differentiated cells; therefore, their damage has irreversible effects. Abnormally expressed miRNAs have been involved in diabetes-induced cell injury and herein we will focus in particular on miRNAs affecting neurons, podocytes, mesangial cells (MCs), and proximal tubular epithelial cells (TECs), as the role of miRNAs in microvascular endothelial cells has been recently reviewed elsewhere [48–50].

Several abnormally expressed miRNAs have been implicated in the pathogenesis of DSPN because they can enhance apoptosis and/or interfere with neuronal regenerative processes. *let-7i* was the most downregulated miRNA in a profiling study on diabetic DRG. Moreover, intranasal injection with a let-7i mimic improved experimental DSPN, and let-7i enhanced both growth and branching of cultured neurons [51], indicating a protective neurotrophic activity. Similarly, *miR-29b* expression was reduced in diabetic DRG and interventions normalizing its levels diminished neuron apoptosis and increased regenerative processes [40]. miR-29b also affects the neuronal component of the diabetic retina. Specifically, miR-29b prevents apoptosis of cultured retinal Müller cells by inhibiting its target gene Sp-1, and thus the downregulation of miR-29b observed in the diabetic retina may favor apoptosis [52]. On the contrary, another member of the miR-29 family *miR-29c* is overexpressed in both DRG neurons and sciatic nerve of diabetic mice and negatively regulates axonal growth by suppressing protein kinase C-iota [53].

Recent studies have explored the role of miRNAs in podocyte damage in diabetes. Nephrin, a major component of the junction connecting foot processes of adjacent podocytes, is crucial to prevent protein leaking, and miR-29, miR-155, and miR-93 have been causally linked to nephrin loss in diabetes. Lin et al. have shown that *miR-29a* was downregulated in the glomeruli from diabetic mice and that miR-29a overexpression attenuated nephrin downregulation, podocyte apoptosis, and proteinuria. The beneficial effect of miR-29a was due to inhibition of histone deacetylase (HDAC4) that causes nephrin deacetylation, ubiquitination, and loss. Not only was HDAC4 a miR-29a target, but also lowered miR-29a expression via an epigenetic mechanism [54], fueling a deleterious vicious cycle. Similarly, a reduction in *miR-93* expression was found in both diabetic glomeruli and podocytes exposed to high glucose [55]. Moreover, diabetic mice with inducible overexpression of miR-93 exclusively in podocytes exhibited significant improvements of albuminuria, nephrin downregulation, foot process effacement, and podocyte loss. miR-93 has an important role in chromatin reorganization by modulating its target MSK2, a histone kinase, and its substrate H3S10, and miR-93 constitutive expression is required to maintain podocyte health. Because miR-93 expression is reduced by hyperglycemia, miR-93 is a critical link between altered metabolism and podocyte epigenetic alterations [56]. Finally, *miR-155* was overexpressed in both human and experimental DN [28, 57], and its deletion enhanced expression of nephrin, acetylated nephrin, and Wilms tumor 1 (WT-1), a marker of podocyte differentiation, through upregulation of suppressor of cytokine signaling 1 (SOCS1) that inhibits the Janus kinase 2 (JAK2)/signal transducer and activator of transcription 1 (STAT1) pathway [57], indicating a deleterious effect of diabetes-induced glomerular miR-155 overexpression.

Besides nephrin downregulation, alterations of the podocyte cytoskeleton have also been implicated in proteinuria, foot process effacement, and podocyte loss. miRNAs abnormally expressed in response to hyperglycemia/TGF-*β*1 appear to contribute. In podocytes, high glucose induces *miR-27a* that activates *β*-catenin signaling by negatively targeting PPAR-*γ*. This leads to increased podocyte mesenchymal transition, disrupted podocyte architectural integrity, downregulated nephrin, and increased podocyte apoptosis. The *in vivo* relevance was proven in experimental DN and miR-27a upregulation confirmed in human diabetic kidney biopsies [58]. In cultured podocytes exposed to high glucose, *miR-29c* suppresses its target gene Spry1, resulting in both abnormal activation of Rho kinase, a key regulator of the podocyte cytoskeleton, and apoptosis. This is in agreement with *in vivo* studies showing miR-29c upregulation in the glomeruli from db/db mice and amelioration of DN in miR-29c knockdown mice [59]. Of interest, linagliptin inhibits the enzyme dipeptidyl peptidase-4 (DPP-4) that degrades miR-29, and the beneficial effect of linagliptin treatment in experimental DN may be partially ascribed to a reduction in miR-29 levels [60]. Finally, *miR-135a* is induced by TGF-*β*1 in cultured podocytes and causes severe podocyte injury and disarray of the podocyte cytoskeleton by downregulating transient receptor potential channel 1 (TRPC1) [61].

Several miRNAs modulate apoptosis in renal and/or retinal cells predominantly by affecting TGF-*β*1 signaling. The expression of *miR-21* is enhanced in both human and experimental DN [42–45], and recent work by Lai JY et al. suggests that both hyperglycemia and TGF-*β*1 induce miR-21 that in turn functions as a feedback inhibitor of TGF-*β*-induced podocyte apoptosis. Consistent with this notion, miR-21 deletion worsened albuminuria, podocyte loss, and renal injury in both diabetic and TGF-*β*1 transgenic mice. *In vitro* studies in podocytes have clarified that the beneficial effect of miR-21 is due to downregulation of proapoptotic target genes (Smad7, TGFR2, and Pdcd4) [42]. Sirtuins (SIRTs) are potent inhibitors of apoptosis and both *miR-195* and *miR-20b* have been shown to affect apoptosis in DMC by suppressing members of the SIRT family. Specifically, miR-195 was overexpressed in both the retina and the kidney of diabetic animals, and both miR-195 and miR-20 mediated high-glucose-induced apoptosis of renal cells by suppressing SIRT1 and SIRT7, respectively [62–65]. *miR-25* is downregulated in both human and experimental DN, and this deficiency has detrimental effects on both MCs and TECs as miR-25 prevents TEC apoptosis by modulating the phosphatase and tensin homolog (PTEN)/Akt pathway and reduces oxidative stress in MCs by targeting NADPH oxidase 4 (Nox4). Consistent with a protective role of miR-25, systemic administration of a miR-25 mimic ameliorated DN [66–68]. Several other miRNAs affect apoptosis; however, given their predominant effect on fibrotic processes, they will be described in the next session.

In the diabetic kidney, MCs undergo proliferation and then hypertrophy, a change in phenotype that preludes to enhanced expression of ECM components. Expression of *miR-34* is enhanced in DN and miR-34 induces MC proliferation by targeting GAS1 [69]. This is in contrast with the effect of miR-34 in retinal cells, where this miRNA reduces cell proliferation by inhibiting LGR4 [70]. MC hypertrophy is induced by *miR-21* through inhibition of its target gene PTEN and activation of the Akt/target of rapamycin complex 1 (TORC1) pathway. This together with the profibrotic effects of miR-21 in MCs may explain reports of amelioration of DN in diabetic miR-21 knockout mice despite the protective antiapoptotic effect of miR-21 on podocytes [43, 71, 72]. Another miRNA implicated in MC hypertrophy is *miR-200b/c*, which is overexpressed in experimental DN. Specifically, TGF-*β*1 induces expression of miR-200b/c that causes downregulation of the PI3K inhibitor FOG2, leading to MC hypertrophy through the PI3K/Akt pathway [73].

Collectively, these data underscore the relevance of miRNA in cell health and the contribution of miRNAs in the pathogenesis of the cellular injury induced by diabetes.

### 4.4. MicroRNAs and Fibrosis in DMC

Excessive deposition of ECM components, mainly collagen and fibronectin, leading to sclerosis, occurs in all DMC, but it is a predominant feature of DN, and thus most of the studies assessing the role of miRNAs on fibrosis were performed in this complication.

**Figure 2:**
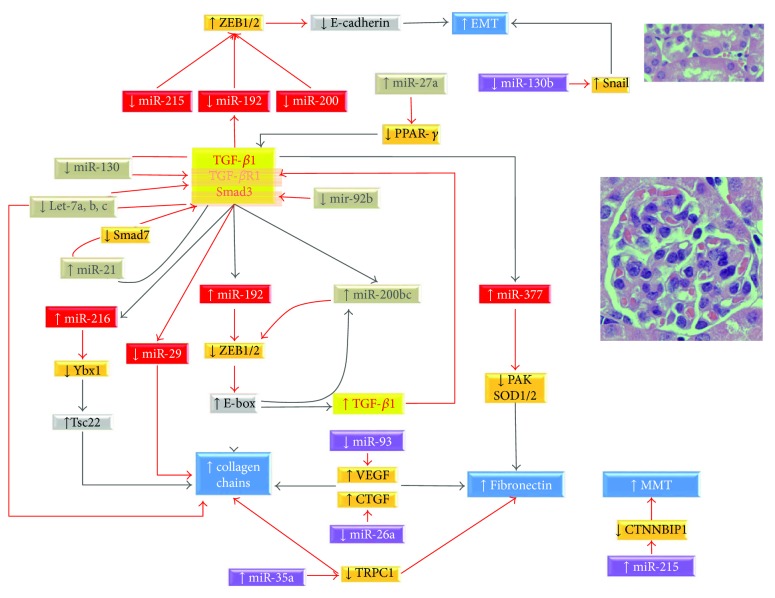
MicroRNAs involved in renal fibrosis in diabetes. MicroRNAs (miRNAs) implicated in glomerular (cream-coloured area) and tubule-interstitial (light purple-coloured area) fibrosis in diabetes. The image shows miRNAs in red boxes that are modulated by TGF-*β*1 and directly control collagen/fibronectin expression, miRNAs in bronze boxes that enhance TGF-*β*1 signaling, and miRNAs in purple boxes that affect fibrosis independently of TGF-*β*1. Target genes are shown in orange boxes. Grey lines indicate induction, while red lines indicate suppression of miRNA expression. EMT: epithelial mesenchymal transition; MMT: mesangial cell to myofibroblast transition; CTNNBIP1: catenin beta interacting protein 1; TGF-*β*1: transforming growth factor-*β*1; TGFB-R1: transforming growth factor type 1 receptor; VEGF: vascular endothelial growth factor; CTGF: connective tissue growth factor.

The prosclerotic cytokine TGF-*β*1, which is released by resident cells in response to diabetes-related insults, acts locally via autocrine/paracrine mechanisms and is a key mediator of fibrotic processes in both the glomeruli and the tubule interstitium. Several studies have thus investigated the complex interplay between miRNAs and TGF-*β*1 on renal fibrosis (Figure 2). TGF-*β*1 induces the expression of profibrotic miR-216 and miR-377, while it represses antifibrotic miR-29 and let-7. Data on the effect of TGF-*β*1 on miR-192 are more conflicting with contrasting results *in vitro* in MCs and TECs and also *in vivo* where miR-192 was found both upregulated and downregulated in experimental DN. This may be due to differences in animal models, disease stages, and/or *in vitro* experimental conditions. In MCs, miR-192 is induced initially through Smad3 signaling and then through a long-lasting epigenetic mechanism involving Ets1 and histone H3 acetylation by Akt-activated p300, and this is consistent with findings in the glomeruli from db/db mice [74]. On the contrary, in TECs, TGF-*β*1 represses miR-192 transcription by decreasing the binding of HNF to the miR-192 gene [75]. HNF expression is restricted to the tubules and this may partially explain the cell specificity of miR-192 expression [76].

The collagen gene has E-box regulatory elements placed in its far upstream region. In MCs exposed to TGF-*β*1 and in glomeruli from diabetic mice, upregulation of *miR-192* and *miR-200b/c* increases Col1a2 and Col4a1 expression by inhibiting the E-box repressors Zeb1 and Zeb2 [77–79]. Consistent with this, a negative correlation between miR-192 and both Zeb1 and Zeb2 expression has been reported in patients with type 2 diabetes [80]. Moreover, diabetic mice either knockout for miR-192 or treated with miR-192 inhibitors had a less severe renal phenotype with amelioration of glomerular hypertrophy, fibrosis, and proteinuria [77, 78, 80]. In MCs, TGF-*β*1 also promotes expression of Col1a2 via upregulation of *miR-216a*. This effect is mediated by posttranscriptional upregulation of Tsc22 by Ybx1, which is a RNA-binding protein targeted by miR-216a. The interaction of Tsc22 with the transcription factor E3 increases Col1a2 expression and, in keeping with this, a significant increase of miR-216 and Tsc22 with parallel Ybx1 downregulation was observed in the glomeruli of diabetic mice [81]. Members of the *miR-29* and *let-7a*, *b*, *c* families suppress the transcription of collagen and have an antifibrotic effect. TGF-*β*1 counteracts this antifibrotic effect by downregulating both miR-29 and miR-7a, b, c [82–89]. Consistent with this, miR-29b overexpression ameliorates DN [38, 82]. Moreover, in a model of advanced DN, an angiotensin receptor blocker that enhanced miR-29 expression reduced both ECM deposition and renal fibrosis [84]. In MCs, TGF-*β*1 can also affect fibronectin expression through *miR-377*. Specifically, TGF-*β*1 enhances expression of miR-377 that increases fibronectin levels by both inhibiting PAK1 and increasing oxidative stress through suppression of superoxide dismutase (SOD)-1/2 genes [90].

As mentioned above, the interplay between miR-192 and TGF-*β*1 differs in TECs. In these cells, TGF-*β*1 induces a downregulation of *miR-200*, *miR-192*, and *miR-215*. As these miRNAs inhibit Zeb1 and Zeb2, this results in E-cadherin downregulation. E-cadherin is not only an epithelial marker, but is also involved in cell-to-cell adhesion, and E-cadherin dysregulation induces phenotypic changes contributing to epithelial mesenchymal transition (EMT) and thus to renal fibrosis [91–95].

A number of miRNAs affect fibrosis by enhancing TGF-*β*1 signaling. For instance, TGF-*β*1 downregulates *let-7a*, *b*, *c* and *miR-130* that are known to suppress expression of the TGF-*β*1 receptor of type 1 [85–89, 96]. The downregulation of *miR-92b* enhances Smad3 expression, while TGF-*β*1-induced *miR-21* represses the expression of inhibitory Smad7 [43–45, 97]. Recently, Kato et al. have described a positive circuit whereby *miR-200b/c* enhances TGF-*β*1 expression in MCs. TGF-*β*1-induced miR-192 represses Zeb1/2, leading to TGF-*β*1 and miR-200b/c expression. In turn, miR-200b/c by inhibiting Zeb1/2 further enhances the effect of miR-192 on TGF-*β*1 [79]. Of interest, miR-200b/c has also been implicated in the fibrotic processes occurring in the diabetic retina. Specifically, miR-200b is increased in human PDR [98] and experimental DR [99] and promotes EMT both *in vitro* and *in vivo* [100]. Finally, diabetes-induced *miR-27a* overexpression promoted fibrosis in both TECs and diabetic rats by targeting PPAR-*γ* and indirectly enhancing TGF-*β*/Smad3 signaling [101].

There are also miRNAs that can affect fibrosis in a TGF-*β*1-independent manner. In podocytes, downregulation of *miR-93* and *miR-26* enhances both fibronectin and collagen expression by reducing the inhibitory effects of miR-93 and miR-26 on VEGF and connective tissue growth factor (CTGF), respectively [102]. In MCs, *miR-135a* induces synthesis of ECM components by inhibiting its target gene TRPC1, and miR-135a deletion restores levels of TRPC1 and reduces production of both fibronectin and collagen type I in experimental DN [103]. *miR-215* contributes to renal fibrosis in DN by suppressing its target gene catenin-*β* interacting protein 1 and thus favoring the transition of MCs to myofibroblasts [104]. *miR-130b* is downregulated in DN and miR-130 overexpression improves tubulointerstitial fibrosis via repression of Snail-induced EMT [105]. Deletion of *miR-146* worsens kidney injury at least in part by targeting the ErbB4 and Notch-1 pathway [33]. In addition, miR-146 can directly control fibronectin expression in retinal cells and *in vivo* a miR-146 mimic normalized fibronectin levels in the retina of diabetic mice [30].

Taken together, these studies prove the crucial role of miRNAs in regulating ECM production and have given us a deeper understanding of the complex mechanisms involved in diabetes-induced fibrotic processes.

## 5. Circulating miRNAs

miRNAs are also present in biological fluids, such as serum, plasma, saliva, urine, milk, and humor vitreous. Although their role is not completely understood, they are likely involved in cell-to-cell communication. In fact, after release by parental cells, circulating miRNAs can translocate into recipient cells in which they regulate gene expression.

miRNAs are very stable in biological fluids as they are either enclosed in microparticles or assembled into complexes that protect them from endogenous RNases. Cells can actively secrete miRNAs packed into exosomes, which are small vesicles released by cells through a tightly regulated active process [106]. Alternatively, miRNAs can be passively released in either apoptotic bodies or microvesicles (MV) by cells exposed to insults [107, 108].

miRNAs are increasingly recognized as a promising biomarker, given the ease with which they can be isolated and their structural stability under different conditions of sample processing and isolation. Data showing that circulating miRNA profiling can be disease-specific representing a molecular signature of the disease support the hypothesis that miRNAs can be valuable clinical biomarkers [109].

Several studies have investigated whether changes of miRNA levels in body fluids are associated with DN and DR, while no study on circulating miRNAs in DSPN is yet available despite evidence of alterations of intracellular miRNAs in DSPN. Clinical miRNA studies differ substantially in study design, patient sample size, type of diabetes, and body fluid analyzed (Table 5). Most studies performed miRNA profiling and validated highly differentially expressed miRNAs, while studies on candidate miRNAs focused on miRNAs known to be altered in relevant tissues.

### 5.1. Circulating MicroRNA and DMC

Based upon profiling results, Wang et al. validated 13 miRNAs and confirmed enhanced serum levels of miR-661, miR-571, miR-770-5p, miR-892b, and miR-1303 in subjects with type 2 diabetes and DMC. Among them, miR-1303, a miRNA involved in autophagy, is of particular relevance as high miR-1303 levels conferred an over threefold increased risk of DMC independently of body mass index and blood pressure [110]. Sebastiani et al. identified miR-31 as an upregulated miRNA in patients with DMC, and this miRNA is likely involved in angiogenesis and vascular permeability based on its predicted target genes (E-selectin, integrin-*α*5, and nitric oxide synthase 1) [111].

### 5.2. Circulating MicroRNAs and Diabetic Retinopathy

Three miRNA profiling studies have been performed on serum samples from patients with and without DR, allowing the identification of potential biomarkers of DR.

In a nested case-control study on two prospective cohorts of patients with type 1 diabetes from the DIRECT-1 trial (PROTECT-1 and PREVENT-1), Zampetaki et al. profiled 29 miRNAs and validated relevant miRNAs. They found that two miRNAs, miR-27b and miR-320a, were associated with the incidence and the progression of DR. Furthermore, proteomic analysis performed in endothelial cells showed that the antiangiogenic protein thrombospondin-1 was a target of both [112].

We have recently performed a miRNA profiling in pooled serum samples from type 1 diabetic patients with and without diabetic complications from the nested case-control study of the EURODIAB PCS. Among the 25 differentially expressed miRNAs, miR-126 was validated. miR-126 levels were lower in cases than in controls and inversely associated with all complications as well as with each complication examined separately. After adjustment for age, sex, A1C, and diabetes duration, a 25% risk reduction was still observed for PDR [113]. A significant decrease in serum miR-126 levels was also reported by another study performed in patients with type 2 diabetes and PDR [114]. Therefore, several studies on both intra- and extracellular miR-126 indicate a key role of this miRNA in PDR.

Qing et al. performed a differential miRNA profiling of serum samples from subjects with DR and identified 3 miRNAs (miR-21, miR-181c, and miR-1179) significantly increased in patients with PDR [115]. A rise in plasma miR-21 levels in patients with type 2 diabetes and DR has also been confirmed by another study [116], and these data are in line with studies showing miR-21 upregulation in experimental DR. Similarly, plasma miR-93 levels were greater in patients with type 2 diabetes and DR compared to those in patients without DR [117].

### 5.3. Circulating MicroRNAs and Diabetic Nephropathy

Pezzolesi et al. performed a prospective study on circulating miRNAs in DN. Specifically, they assessed if the circulating levels of 5 miRNAs, which are under the control of TGF-*β*, predicted the development of ESRD during follow-up in patients with type 1 diabetes and proteinuria but normal renal function at baseline. They found that let-7c-5p and miR-29a-3p were associated with an over 50% reduction of the risk of rapid progression to ESRD, while let-7b-5p and miR-21-5p were associated with a 2.5-fold increase in ESRD risk independently of HbA1c and other confounders [118]. This finding is of particular relevance given the lack of clinical biomarkers to predict ESRD and supports the hypothesis that measurement of miRNA circulating levels may be of relevance in clinical practice to identify subset of patients at high risk.

Recently, Zhou et al. performed a miRNA microarray assay in patients with type 2 diabetes and found that let-7a was differentially expressed in patients with DN as also confirmed by PCR. In addition, they discovered that the rs1143770 variant of the let-7a-2 gene was associated with an increased risk for DN [119].

Other studies focused on selected miRNAs and reported significant changes in the circulating levels of miR-217, 21, 29a, 192, 130, and 126 in type 2 diabetes patients with and without albuminuria [120–123]. However, given the cross-sectional design of these studies, it is unknown whether these miRNAs can help predicting either the development or the progression of DN. Moreover, levels of miRNAs were often correlated with HbA1c, and it is thus unclear whether these changes in miRNA levels were specific of DN or simply mirrored worse metabolic control in patients with albuminuria.

### 5.4. Urinary miRNAs in DN

Urine is another body fluid that has been used to identify miRNA biomarkers in DN. Two studies on the urinary sediment from patients with chronic kidney diseases showed that miR-15 and miR-192 levels were lower in patients with DN than those in subjects with other kidney diseases [124, 125], suggesting that circulating miRNA may be of practical value in the differential diagnosis of renal diseases.

Other groups have measured free miRNAs into urine. A recent study found that urinary miR-2861, miR-1915-3p, and miR-4532 levels were enhanced in patients with DN and correlated with both renal function and tubulointerstitial injury [126]. Argyropoulos et al. performed a miRNA profiling in patients with type 1 diabetes and worsening stages of albuminuria and found 27 differentially expressed urinary miRNAs [127]. Importantly, the same group also carried out a prospective study assessing the expression of 723 urinary miRNAs in patients with type 1 diabetes who were normoalbuminuric at baseline. Eighteen miRNAs were found associated with microalbuminuria development, and 9 of them were used to define a miRNA signature for microalbuminuria [128].

More recently, several studies have measured/profiled miRNAs enclosed in urinary exosomes/MVs. In type 1 diabetes, we reported that urinary exosomes from patients with microalbuminuria were enriched in miR-130a and miR-145, while their content in miR-155 and miR-424 was reduced. Of interest, exosomes either released by MCs exposed to high glucose or isolated from the urine of animals with DN were also enriched in miR-145 [129]. Other studies on urinary exosomes/MVs were performed in patients with type 2 diabetes. Delić et al. identified 16 differentially expressed miRNAs in urinary exosomes from patients with DN and confirmed that exosomal miR-320 and miR-6068 content was greater in patients with DN [130]. Another study showed increased levels of miR-133b, miR-342, and miR-30a in patients with DN [131]. Finally, a study performed on urinary MVs from patients with type 2 diabetes and different degrees of albuminuria revealed that miR-192, miR-194, and miR-215 levels were specifically increased in patients with microalbuminuria and that there was a correlation between miR-192 and TGF-*β*1 levels as expected based on their close relationship in renal tissue [132].

## 6. Future Perspective and Limits of This Line of Research

**Figure 3:**
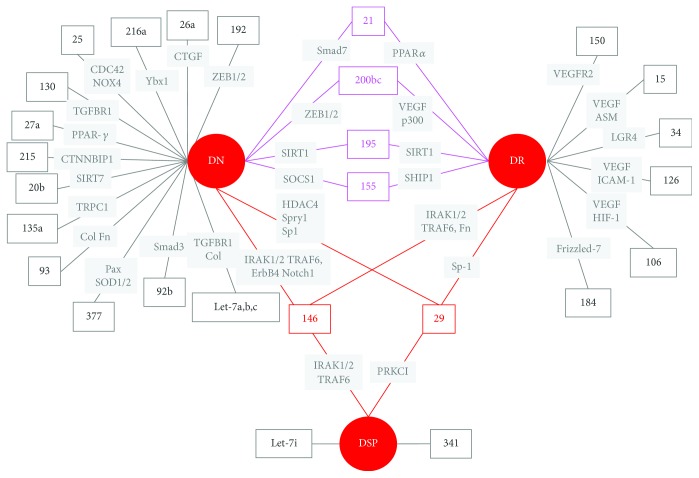
MicroRNA involved in diabetes microvascular complications. miRNAs and miRNA targets (in italics) abnormally expressed in diabetic nephropathy (DN), diabetic retinopathy (DR), and diabetic neuropathy (DSP) are shown. Pink lines connect miRNAs involved in both DN and DR. Red lines connect miRNAs involved in all diabetes microvascular complications.

In the last decade, much progress has been made in our understanding of miRNA role in the pathogenesis of DMC. Today, we know that several miRNAs are deregulated and play a major pathogenic role in DMC. Moreover, vascular complications of diabetes share, at least in part, insults and underlying pathogenic mechanisms, and some miRNAs have been implicated in multiple diabetes complications (Figure 3). The best example is miR-146 that is involved in all DMC and also in the pathogenesis of atherosclerosis [133]. Another miRNA that has attracted much attention in multiple areas of diabetes research is miR-126. Besides playing a role as both mediator and marker of PDR, this miRNA is also a biomarker of the risk of developing type 2 diabetes [134, 135] and has been implicated in the pathogenesis of CVD [136]. These overlaps may be advantageous as therapies targeting single miRNAs may have beneficial effects on other vascular beds.

In other fields, such as oncology, research on miRNAs has already moved from gaining a better knowledge of miRNA involvement in pathogenic mechanisms to the use of miRNAs as targets for intervention. Gene therapy designed to modulate miRNA expression can be applied to either increase or decrease miRNA levels in order to obtain desirable clinical outcomes. However, delivery to relevant tissues/organs is still a major issue. Chemically modified oligonucleotides, sponges, MV, viruses, and gold nanoparticles have been tested for both effectiveness and specificity of delivery; however, many problems still need to be addressed. Besides delivery, treatments targeting single miRNAs can affect many genes, as a single miRNA controls the expression of multiple mRNAs, possibly causing undesired off-target effects, and this is an important pitfall of potential new therapies manipulating miRNAs.

Available data on circulating miRNAs in DMC are still scarce, but they are likely to increase in the near future as the number of studies assessing miRNAs as biomarkers is growing exponentially. However, there are several limitations in this area of research. First, reproducibility of results in other series of patients and using different methodologies is very poor and this underscores the importance of a coordinate effort to standardize both collection and analyses of biological samples for miRNA biomarker discovery and to use appropriate and homogenous endogenous controls. Second, circulating miRNAs derived from damaged/necrotic cells are a possible confounder and measurement of miRNAs enclosed in exosomes/MV may be a preferable option. However, the procedure of exosome/vesicle isolation is both time-consuming and expensive, making miRNAs less attractive as biomarkers. Third, despite the growing number of studies measuring miRNAs in body fluids, there is relatively little knowledge on how environmental variables, including drug therapies, affect circulating miRNA levels in normal subjects, and this can significantly hamper our understanding of the significance of miRNA changes in pathological conditions. Therefore, studies covering this gap in knowledge are desperately needed. Fourth, prospective studies in longitudinal cohorts are required to evaluate whether potential novel biomarkers of DMC have a predictive value, and their addition to currently available clinical markers and scores improves identification of subgroup of patients at high risk of DMC development/progression.

Most of the research on miRNA biomarkers in DMC has been hypothesis driven and non-hypothesis-driven research is still in its infancy in the field of diabetes. However, both improved availability of biobanks that store samples from clinical cohorts, and increasing experience with the use of both omic technologies and biostatistics is likely to change this scenario in the next future. “Molecular signatures,” obtained using an open omic approach, are likely to outperform research based on individual biomarkers as single biomarkers can hardly reflect the biological complexity of the underlying microvascular injury.

## 7. Conclusions

Lately, our understanding of the importance of miRNAs in the pathogenesis of DMC has grown substantially, and intervention studies in experimental animals indicate that treatments targeting miRNAs can be beneficial. Moreover, there is increasing evidence for a potential role of miRNAs as clinical biomarkers of DMC. Discovery of new set of miRNA biomarkers might help to guide diagnostic and therapeutic decisions and to facilitate the implementation of personalized medicine into the clinical setting. However, novel miRNA biomarkers must be rigorously validated in adequately powered, prospective, independent clinical studies prior to implementation in clinical practice.

## Figures and Tables

**Table 1:** miRNA profiling studies in diabetic microvascular complications.

DMC	Study design	Source		Number of ΔmiRNAs	Selected differentially expressed miRNA	Ref
DR	STZ-DM rats versus controls	Retinas	Δ86		↑ 31, 31^∗^, 34b-3p, 34c, 184,199a, 200a, 200b, 205, 223, 335-3p, 378^∗^, 488, 574-3p ↓ 20b, 499, 690 (*17 PCR-validated miRNAs*)	[22]
		REC	Δ120		↑ *15b*, *19b*, *21*, *31*, *132*, *142-3p*, *146a*, *155*, *339-5p*, *342-3p*, *450a* ↓ *20b-5p*, *29c*, *181c*, *136*^∗^, *376c* *(16 PCR-validated miRNAs)*	
	db/db mice versus controls	Retinas	Δ6		↑ 21, 31, 132, 184, 379, 322 ↓ 29b, 294^∗^, 376b, 690, 712 *(>2-fold increase)*	[41]
	DM2 patients versus controls	REC	↑ 60, ↓ 16		↑ 133 ↓ 10a, 10b, 15a *(4 PCR-validated miRNAs)*	[21]

DSPN	STZ-DM mice versus controls	DRG	↑ 74 ↓ 68		↓ let-7i, 103, 16, 107, 130a, 30a, 29a, 138, 27a, 27b, let7g, 30b, let7f, 34a, 338-3p ↑ 191, 466-5p, 149^∗^, 341^∗^ *(>1.5 fold-change)*	[51]
	STZ-DM mice versus controls	Spinal dorsal horn	↑ 21 ↓ 21		↑ 184-5p ↓190a-5p *(2 PCR-validated miRNAs)*	[137]

DN	DN FSGS IgAN MPGN Controls	Human glomeruli	DN: Δ18 FSGS: Δ12 IgAN: Δ2 MPGN: Δ17 versus controls		DN versus controls: ↑ 29a, 23a, 214, 21, 5585, 589, 150, 4286 ↓ 486-3p, 486-5p, 1180, 4301, 30a-3p, 30c, 148a, 30a-5p, 3184, 423 DN versus FSGS: ↑ 24, 146a DN versus MPGN:↑ 146a, 146b; ↓ 671	[37]
	DM2 patients versus healthy controls	Kidney	↑ 32 ↓ 39		↑ 146a, 155 *(2 PCR-validated miRNAs)*	[28]
	db/db mice versus controls	Glomeruli	↓ 45	↓ 5	↓ 92a, 92b, 93, 140, 191	[55]
	HG versus NG	Podocytes	↓ 32			
		GEC	↓ 86			

DMC: diabetic microvascular complications; DM: diabetes; DM2: type 2 diabetes; STZ-DM: streptozotocin-induced diabetes; DR: diabetic retinopathy; DN: diabetic nephropathy; DSPN: diabetic peripheral symmetric polyneuropathy; HG: high glucose; NG: normal glucose; FSGS: focal segmental glomerulosclerosis; MPGN: membranoproliferative glomerulonephritis; REC: retinal endothelial cells; GEC: glomerular endothelial cells; DRG: dorsal root ganglia; Δ: differentially expressed miRNAs. ^∗^miRNA nomenclature.

**Table 2:** MicroRNAs Involved in Diabetic Retinopathy.

miRNA	Source	Model	miRNA levels	Putative targets	Pathogenic role	Reference
126	Retinas	STZ-DM rats, OIR mice	↓	*VCAM-1 VEGF*	↑ angiogenesis	[15–17]
200b	Retinas	STZ-DM rats and DM patients	↓	*VEGF*	↑ angiogenesis	[20]
	Vitreous	Patients with PDR, Akita-DR mice, STZ-DM mice	↑	*Oxr1* *Snail1* *Smad2* *p300*	↑ EMT	[98–100]
15a	Retinas RPE cells	STZ-DM mice and STZ-DM rats, HRPE cells	↓	*VEGF-A* *ASM*	↑ angiogenesis, ↑ inflammation, ↑ lipotoxicity	[21]
150	Retinas	STZ-DM rats HFD-DM mice (WT and miR-150^−/−^)	↓	*VEGFR2*	↑ angiogenesis	[22, 23]
184	Retinas	OIR mice	↓	*Frizzled-7*	↑ angiogenesis	[24]
155	Retinas	OIR mice	↑	*SHIP1*	↑ angiogenesis	[26]
146a	Retinas	STZ-DM rats	↓	*CARD10* *IRAK1/2 TRAF6*	↑ inflammation	[22, 34]
	Retinas	STZ-DM rats and db/db mice	↓	*Fibronectin*	↑ fibrosis	[30]
21	Retinas	STZ-DM rats and db/db mice	↑	*PPARα*	↑ inflammation	[22, 41]
195	Retinas	STZ-DM rats	↑	*SIRT1*	↑ apoptosis	[62]
29b	Retinas	STZ-DM mice, STZ-DM rats	↓	*Sp1*	↑ apoptosis	[39, 52]

RPE: retinal pigment epithelial cells; STZ: streptozotocin; OIR: oxygen-induced retinopathy; DM: diabetes; PDR: proliferative diabetic retinopathy; HFD: high-fat diet; WT: wild type; EMT: epithelial mesenchymal transition.

**Table 3:** MicroRNAs involved in diabetic neuropathy.

miRNA	Source	Model	miRNA levels	Putative targets	Pathogenic role	Reference
146	Sciatic nerve	db/db mice	↓	*IRAK1/2 TRAF6*	↑ inflammation, apoptosis	[31, 32]
		STZ-DM rats	↑	*IRAK1/2 TRAF6*	Dysfunctional NF-*к*B-miR-146a negative feedback loop	[27]
let-7i	DRG neurons	STZ-DM mice	↓	*—*	↓ neurotrophism regeneration	[51]
29b	DRG neurons	STZ-DM rats	↓	*Sp1 (?)*	↑ apoptosis, ↓ regeneration	[40]
29c	DRG neurons, sciatic nerve, and foot pad tissues	db/db mice	↑	*PRKCI*	↓ axonal growth	[53]
341	DRG neurons	STZ-DM mice	↑	*—*	Unknown	[51]

DRG: dorsal root ganglia; STZ: streptozotocin; DM: diabetic.

**Table 4:** MicroRNA involved in diabetic nephropathy.

miRNAs	Source	Model	miRNA levels	Putative targets	Pathogenic role	Reference
21	Glomeruli Kidney	DM2 patients with albuminuria, STZ-DM mice	↑	*SMAD7*, *TGF-βR2* *PDCD4*, *Col4a1*, *TIMP3*	↓ fibrosis, ↓ podocyte damage	[42]
	Kidney	db/db mice KK/Ay mice	↑	*SMAD7*	↑ fibrosis, ↑ inflammation	[44, 45]
	Kidney	OVE26 mice	↑	*PTEN*	↑ fibrosis	[43]
	Glomeruli	db/db mice	↓	*PTEN*	↑ fibrosis	[72]
25	Kidney	DM2 patients with DN STZ-DM rats STZ-DM mice and db/db mice	↓	*PTEN* *NOX4* *CDC42*	↑ oxidative stress, ↑ apoptosis, ↑ fibrosis, ↑ podocyte injury	[66–68]
26a	Glomeruli	DM2 patients with DN STZ-DM mice	↓	*CTGF*	↑ fibrosis	[102]
27a	Glomeruli Kidney	DM2 patients with DN STZ-DM rats	↑	*PPAR-γ*	↑ podocyte damage, ↑ fibrosis	[58, 101]
29a	Glomeruli	STZ-DM mice	↓	*HDAC4*	↑ podocyte injury, ↑ fibrosis	[54]
29b	Kidney	db/db mice	↓	*SP1*	↑ fibrosis, ↑ inflammation	[38]
29c	Glomeruli	db/db mice	↑	*SPRY1*	↑ fibrosis, ↑ podocyte injury	[59]
34a	Glomeruli	db/db mice	↑	*GAS1*	Glomerular hypertrophy	[69]
93	Glomeruli Kidney	db/db mice DM patients with DN	↓	*MSK2/VEGF*	↑ VEGF, podocyte damage	[55, 56]
130b	Glomeruli	STZ-DM mice	↓	*TGF-βR1*	↑ fibrosis	[89]
	Kidney	DM2 patients and STZ-DM rats	↓	*SNAIL*	↑ EMT	[105]
135a	Kidney	DM patients with DN and db/db mice	↑	*TRPC1*	↑ fibrosis	[103]
146a	Kidney	DM2 patients with DN, STZ-DM rats and DM2 rats STZ-DM mice and db/db mice	↑	*IRAK1*, *TRAF6*	↓ inflammation, ↓ fibrosis	[28]
		DM2 patients and ob/ob mice	↓	*ERB-B4*, *NOTCH-1*	↑ podocyte injury	[33]
155	Kidney	DM2 patients with DN, STZ-DM rats and DM2 rats STZ-DM mice	↑	*SOCS1*	↓ inflammation, ↑ podocyte damage	[28, 57]
192	Kidney	Patients with advanced DN ApoE-KO DM mice	↓	*ZEB1/2*	↑ EMT, ↑ fibrosis (tubule-interstitial)	[91, 92]
	Glomeruli Kidney	STZ-DM mice and db/db mice DM2 patients with early DN	↑	*ZEB1/2*	↑ fibrosis (glomeruli)	[77, 78, 80]
195	Glomeruli Kidney	STZ-DM mice (proteinuric)	↑	*BCL2*	↑ podocyte damage/apoptosis	[63]
	Kidney	Early DN mice	↓	*BCL2*	↑ mesangial cell proliferation	[64]
215	Glomeruli Kidney	db/db mice	↑	*CTNNBIP1*	↑ EMT	[104]
	Kidney	ApoE-KO STZ mice	↓	*ZEB2*	↑ EMT, ↑ fibrosis	[87]
216a	Glomeruli	STZ-DM mice and db/db mice	↑	*YBX1*	↑ fibrosis	[81]
200b	Kidney	ApoE KO STZ mice	↓	*ZEB1/2*, *FN*	↑ EMT	[93–95]
200b/c	Glomeruli	STZ-DM mice and db/db mice	↑	*FOG2*, *ZEB1*	Glomerular hypertrophy	[73, 79]
377	Kidney	STZ-DM mice and NOD mice	↑	*PAK1*, *SOD2*	↑ fibrosis	[90]
let-7a	Kidney	DM2 patients with early DN STZ-DM rats	↓	*UHRF1* *TGF-βR1*	↑ fibrosis	[88, 96]
let-7/7b	Glomeruli Kidney	ApoE-KO STZ mice	↓	*Col1a2*, *Col4a1* *TGF-βR1*	↑ fibrosis	[85, 86]

STZ: streptozotocin; DM: diabetes; DN: diabetic nephropathy; KO: knockout; EMT: epithelial mesenchymal transition.

**Table 5:** Circulating microRNAs in diabetic microvascular complications.

miRNA	Type of DM	Study design	Study population	Profiling	Source	Significant comparisons-details	Reference
*Diabetes microvascular complications*							
↑ 661 ↑ 571 ↑ 770-5p ↑ 892b ↑ 1303	DM2	Case Control	DMC+ (*n* = 92) DMC− (*n* = 92) Controls (*n* = 92)	Yes	Serum	DM versus controls DMC+ versus DMC−	[110]
↑ 31	DM2	Case Control	DMC (*n* = 12) DM-CVD (*n* = 12) DM (*n* = 12)	Yes	Serum	DMC versus others	[111]
*Diabetic retinopathy*							
↓ 27b ↑ 320a	DM1	Nested Case Control	Incidence of DR (*n* = 62) Progression of DR (*n* = 93) Controls (*n* = 145)	Yes	Serum	27b: OR: incidence of DR 0.57 (0.40, 0.82); 320: OR: incidence of DR 1.57 (1.07, 2.31), OR: progression of DR 1.43 (1.05, 1.94)	[112]
↓ 126	DM1	Nested Case Control	Complications+ (*n* = 312) Complications− (*n* = 143)	Yes	Serum	OR: PDR 0.75 (0.59–0.95)	[113]
↑ 21 ↑ 181c ↑ 1179	DM2	Case Control	PDR (*n* = 90) NPDR (*n* = 90) Controls (*n* = 20)	Yes	Serum	PDR versus NPDR	[115]
↓ 126	DM2	Case Control	PDR (*n* = 39) NPDR (*n* = 42) DR− (*n* = 44) Controls (*n* = 59)	No	Serum	PDR versus DR−	[114]
↑ 93	DM2	Case Control	DR+ (*n* = 75) DR− (*n* = 65) Controls (*n* = 127)	No	Plasma	DR+ versus DR−	[117]
↑ 21	DM2	Case Control	PDR (*n* = 51) NPDR (*n* = 73) DR− (*n* = 65) Controls (*n* = 115)	No	Plasma	PDR and NPDR versus DR−	[116]
↑ 23a ↑ 320a-b	DM2	Case Control	PDR (*n* = 4) ME (*n* = 4)	Yes	Vitreous/ serum	PDR versus ME	[138]
*Diabetic nephropathy*							
↓ let-7c ↓ 29a ↑ let-7b ↑ 21	DM1	Prospective: rapid progression to ESRF	Macro RP (*n* = 38) Macro NP (*n* = 38) Normo (*n* = 40)	No	Plasma	let-7c: OR: 0.23 (0.10, 0.53) 29a: OR: 0.39 (0.20, 0.76) let-7b: OR: 2.38 (1.31, 4.0) 21: OR: 5.87 (1.68, 20.46)	[118]
↑ 217	DM2	Case Control	Normo (*n* = 186) Micro (*n* = 169) Macro (*n* = 140) Controls (*n* = 195)	No	Serum	DM2 versus controls Micro versus normo Macro versus micro	[121]
↑ 21 ↑ 29a ↑ 192	DM2	Case Control	Macro (*n* = 21) Micro (*n* = 17) Normo (*n* = 12)	No	Serum	Macro versus micro and normo CKD+ (*n* = 18) versus CKD− (*n* = 32)	[120]
↓ 130	DM2	Case Control	Normo (*n* = 137) Micro (*n* = 122) Macro (*n* = 68) Controls (*n* = 131)	No	Serum	Macro versus micro and normo Micro versus normo Controls versus others	[123]
↓ let-7a	DM2	Case Control	DN− (*n* = 104) DN+ (*n* = 108) Controls (*n* = 62)	Yes	Blood	DN+ versus DN−	[119]
↓ 126	DM2	Case Control	Normo (*n* = 52) DM2-micro (*n* = 29) DM2-macro (*n* = 21) Controls (*n* = 50)	No	Blood	Micro-macro versus controls and normo Macro versus micro	[122]
*Urinary miRNA in diabetic nephropathy*							
↓ 15	DM2	Case Control	CKD-IgAN (*n* = 17) CKD-DN (*n* = 17) CKD-HTN (*n* = 22)	Yes	Urinary sediment	DN versus others CKD	[124]
↓ 192	DM2	Case Control	DN (*n* = 20) MCN/FGS (*n* = 21) MGN (*n* = 23) Controls (*n* = 10)	No	Urinary sediment	DN versus others	[125]
↓ 2861 ↓ 1915-3p ↓ 4532	DM2	Case Control	DN+ (*n* = 74) DN− (*n* = 71)	Yes	Urine	DN+ versus DN−	[126]
↓ 323b-5p ↑ 122-5p ↑ 429	DM1	Case Control	Normo (*n* = 10) Intermittent micro (*n* = 10) Persistent micro (*n* = 10) Macro (*n* = 10)	Yes	Urine	Persistent versus intermittent micro	[127]
105, 1972, 28, 30b, 363, 424, 486, 495, 548o	DM1	Prospective: micro onset	Normo (*n* = 27)	Yes	Urine	9 miRNAs: molecular miRNA signature for microalbuminuria	[128]
29a	DM2	Case Control	Macro/micro (*n* = 42) Normo (*n* = 41)	No	Urine	Macro/micro versus normo	[139]
↑ 130a ↑ 145 ↓ 155 ↓ 424	DM1	Case Control	Micro (*n* = 12) Normo (*n* = 12)	Yes	Urinary exosomes	Micro versus normo	[129]
↑ 320c ↑ 6068	DM2	Case Control	DN+ (*n* = 8) DN− (*n* = 8) Controls (*n* = 8)	Yes	Urinary exosomes	DN+ versus DN− and controls	[130]
↑ 133b ↑ 342 ↑ 30a	DM2	Case Control	Macro (*n* = 44) Micro (*n* = 66) Normo (*n* = 56) Controls (*n* = 54)	No	Urinary exosomes	Macro and micro versus normo (133b only macro versus micro)	[131]
↑ 192 ↑ 194 ↑ 215	DM2	Case Control	Normo (*n* = 30) Micro (*n* = 30) Macro (*n* = 20) Controls (*n* = 10)	No	Urinary MV	Micro versus normo, controls, and macro	[132]

DM1: type 1 diabetes; DM2: type 2 diabetes; ESRF: end-stage renal failure; OR: odds ratio; DMC: diabetic microvascular complications; CVD: cardiovascular disease; DR: diabetic retinopathy; PDR: proliferative diabetic retinopathy; NPDR: nonproliferative diabetic retinopathy; Micro: microalbuminuria; Normo: normoalbuminuria; Macro: macroalbuminuria; DN: diabetic nephropathy; CKD: chronic kidney disease; RP: rapid progressors; NP: nonprogressors; IgAN: IgA nephropathy; HTN: hypertensive nephrosclerosis; MCN: minimal change nephropathy; FGS: focal glomerulosclerosis; MGN: membranous glomerulonephropathy; MV: microvesicles; ME: macular edema.
